# Supplementary material for: Neural coding of choice and outcome are modulated by uncertainty in orbitofrontal but not secondary motor cortex
Source: Nat Commun. 2025 Oct 8;16:8931. doi: 10.1038/s41467-025-63866-5 (PMC12508120; doi:10.1038/s41467-025-63866-5)
Supplement: Supplementary file 2 — Reporting Summary [file 41467_2025_63866_MOESM2_ESM.pdf]

nature portfolio

Corresponding author(s): Juan Luis Romero-Sosa
Alex Yeghikian
Andrew Wikenheiser
Tad Hugh Blair
Alicia Izquierdo

Last updated by author(s): Jul 21, 2025

Reporting Summary

Nature Portfolio wishes to improve the reproducibility of the work that we publish. This form provides structure for consistency and transparency in reporting. For further information on Nature Portfolio policies, see our Editorial Policies and the Editorial Policy Checklist.

Statistics

For all statistical analyses, confirm that the following items are present in the figure legend, table legend, main text, or Methods section.

n/a	Confirmed	
☐	☒	The exact sample size (*n*) for each experimental group/condition, given as a discrete number and unit of measurement
☐	☒	A statement on whether measurements were taken from distinct samples or whether the same sample was measured repeatedly
☐	☒	The statistical test(s) used AND whether they are one- or two-sided<br>*Only common tests should be described solely by name; describe more complex techniques in the Methods section.*
☐	☒	A description of all covariates tested
☐	☒	A description of any assumptions or corrections, such as tests of normality and adjustment for multiple comparisons
☐	☒	A full description of the statistical parameters including central tendency (e.g. means) or other basic estimates (e.g. regression coefficient) AND variation (e.g. standard deviation) or associated estimates of uncertainty (e.g. confidence intervals)
☐	☒	For null hypothesis testing, the test statistic (e.g. *F*, *t*, *r*) with confidence intervals, effect sizes, degrees of freedom and *P* value noted<br>*Give P values as exact values whenever suitable.*
☒	☐	For Bayesian analysis, information on the choice of priors and Markov chain Monte Carlo settings
☐	☒	For hierarchical and complex designs, identification of the appropriate level for tests and full reporting of outcomes
☐	☒	Estimates of effect sizes (e.g. Cohen's *d*, Pearson's *r*), indicating how they were calculated

Our web collection on statistics for biologists contains articles on many of the points above.

Software and code

Policy information about availability of computer code

Data collection	Behavioral data was collected using ABET II Software for Operant Control (Commercial, Lafayette Instruments).<br>Calcium imaging was recorded using Miniscope-DAQ-DT-Software version 1.11 (Open Source, Aharoni Lab)
Data analysis	Behavioral Data Analysis, Statistical Analysis, and Plotting was all done using Matlab R2023b and Python version 3.10<br>Calcium imaging post-processing was done on Python using CaImAn version 1.11.14

For manuscripts utilizing custom algorithms or software that are central to the research but not yet described in published literature, software must be made available to editors and reviewers. We strongly encourage code deposition in a community repository (e.g. GitHub). See the Nature Portfolio guidelines for submitting code & software for further information.

Data

Policy information about availability of data

All manuscripts must include a data availability statement. This statement should provide the following information, where applicable:

- Accession codes, unique identifiers, or web links for publicly available datasets
- A description of any restrictions on data availability
- For clinical datasets or third party data, please ensure that the statement adheres to our policy

All the Data and the custom scripts are available using Code Ocean during review. The source data are included in the paper and will then be available on the lab G-Node and the custom scripts will be available on the lab GitHub after the paper has been published.

Research involving human participants, their data, or biological material

Policy information about studies with human participants or human data. See also policy information about sex, gender (identity/presentation), and sexual orientation and race, ethnicity and racism.

Reporting on sex and gender	N/A
Reporting on race, ethnicity, or other socially relevant groupings	N/A
Population characteristics	N/A
Recruitment	N/A
Ethics oversight	N/A

Note that full information on the approval of the study protocol must also be provided in the manuscript.

Field-specific reporting

Please select the one below that is the best fit for your research. If you are not sure, read the appropriate sections before making your selection.

☐ Life sciences ☒ Behavioural & social sciences ☐ Ecological, evolutionary & environmental sciences

For a reference copy of the document with all sections, see nature.com/documents/nr-reporting-summary-flat.pdf

Behavioural & social sciences study design

All studies must disclose on these points even when the disclosure is negative.

Study description	We tested rodents on a flexible decision making task where the rats have to adapt to a changing reward environment. Meanwhile, we recorded neuronal activity in Secondary Motor Cortex (M2) or Orbitofrontal Cortex (OFC) to better understand the involvement of each brain region during flexible learning. We also inhibited neuronal activity in a separate cohort of animals to understand the causal involvement of these brain regions in flexible learning under uncertainty.
Research sample	We used Long Evans rats, both males and females.
Sampling strategy	For sampling procedures we sampled a random number of neurons that happened to be transfected with GCamp6f (a calcium activity indicator) to perform all of our neuronal activity analysis. Similarly for the inhibition study there was a random number of neurons that happened to be transfected with inhibitory DREADDs (a chemogenetic inhibitor). We did not perform any statistics to predetermine the number of animals we should sample from but we have used what is standard practice based on our previous published work and it is a comparable sample size to other published articles by the Nature publishing group.
Data collection	Data was either collected by the operant chamber software (in the case of behavior) or via a miniscope (in the case of calcium activity). The rats were alone inside of the operant chambers. The researchers that were performing the experiments were not blinded to the experimental conditions and the hypotheses during data collection.
Timing	Data was collected in 4 different cohorts. For the calcium data the first cohort's data was collected between 02/25/2023 and 03/03/2023, the data for the second cohort was collected between 12/06/2023 and 12/14/2023. For the chemogenetic experiment the first cohort of data was collected between 08/28/2024 and 09/06/2024 and between 11/20/2024 and 11/29/2024 for the second cohort.
Data exclusions	In the chemogenetic experiment we excluded data from 6 animals because they did not have good transfection in the targeted regions. The inhibitory DREADDs were either only present in one hemisphere for the area of interest or there was no expression of

	the inhibitory DREADDs at all.
Non-participation	No Participants declined participation; N/A
Randomization	For the chemogenetic experiment we performed a within-subject control where the rats received one day of active drug (CNO) that would inhibit the area of interest and the other day they were administered vehicle (VEH). These rats were randomly assigned to these groups.

Reporting for specific materials, systems and methods

We require information from authors about some types of materials, experimental systems and methods used in many studies. Here, indicate whether each material, system or method listed is relevant to your study. If you are not sure if a list item applies to your research, read the appropriate section before selecting a response.

Materials & experimental systems

n/a	Involved in the study	
☒	☐	Antibodies
☒	☐	Eukaryotic cell lines
☒	☐	Palaeontology and archaeology
☐	☒	Animals and other organisms
☒	☐	Clinical data
☒	☐	Dual use research of concern
☒	☐	Plants

Methods

n/a	Involved in the study	
☒	☐	ChIP-seq
☒	☐	Flow cytometry
☒	☐	MRI-based neuroimaging

Animals and other research organisms

Policy information about studies involving animals; ARRIVE guidelines recommended for reporting animal research, and Sex and Gender in Research

Laboratory animals	We used Long Evans rats.
Wild animals	We did not use wild animals.
Reporting on sex	Every experiment in this project involved both males and females so we do not think that the findings apply to only one sex.
Field-collected samples	N/A
Ethics oversight	This project was performed under the approval of UCLA's Animal Research Committee, which is the university's Institutional Animal Care and Use Committee (IACUC).

Note that full information on the approval of the study protocol must also be provided in the manuscript.

Plants

Seed stocks	N/A
Novel plant genotypes	N/A
Authentication	N/A
